# Dietary Intake Mediates Ethnic Differences in Gut Microbial Composition

**DOI:** 10.3390/nu14030660

**Published:** 2022-02-04

**Authors:** Kirra Borrello, Unhee Lim, Song-Yi Park, Kristine R. Monroe, Gertraud Maskarinec, Carol J. Boushey, Lynne R. Wilkens, Timothy W. Randolph, Loïc Le Marchand, Meredith A. Hullar, Johanna W. Lampe

**Affiliations:** 1 Molecular Cell Biology and Public Health, University of Hawaii at Manoa (UHM), Honolulu, HI 96822, USA; kirrab@hawaii.edu; 2 Population Sciences in the Pacific Program, University of Hawaii Cancer Center, UHM, Honolulu, HI 96813, USA; SPark@cc.hawaii.edu (S.-Y.P.); gertraud@cc.hawaii.edu (G.M.); CJBoushey@cc.hawaii.edu (C.J.B.); lynne@cc.hawaii.edu (L.R.W.); loic@cc.hawaii.edu (L.L.M.); 3 Keck School of Medicine, University of Southern California, Los Angeles, CA 90007, USA; KMonroe@usc.edu; 4 Fred Hutchinson Cancer Research Center, Seattle, WA 98109, USA; trandolp@fredhutch.org (T.W.R.); MHullar@fredhutch.org (M.A.H.); JLampe@fredhutch.org (J.W.L.)

**Keywords:** diet, fecal microbiome, mediation, race/ethnicity

## Abstract

Background: The human gut microbiome (GM) has been observed to vary by race/ethnicity. Objective: Assess whether racial/ethnic GM variation is mediated by differences in diet. Design: Stool samples collected from 2013 to 2016 from 5267 healthy Multiethnic Cohort participants (age 59–98) were analyzed using 16S rRNA gene sequencing to estimate the relative abundance of 152 bacterial genera. For 63 prevalent genera (>50% in each ethnic group), we analyzed the mediation of GM differences among African Americans, Japanese Americans, Latinos, Native Hawaiians, and Whites by overall diet quality (Healthy Eating Index score (HEI-2015)) and intake amounts of 14 component foods/nutrients assessed from 2003 to 2008. For each significant mediation (*p <* 1.3 × 10^−5^), we determined the percent of the total ethnicity effect on genus abundance mediated by the dietary factor. Results: Ethnic differences in the abundance of 12 genera were significantly mediated by one or more of eight dietary factors, most frequently by overall diet quality and intakes of vegetables and red meat. Lower vegetable intake mediated differences in *Lachnospira* (36% in African Americans, 39% in Latinos) and *Ruminococcus-1* (−35% in African Americans, −43% in Latinos) compared to Native Hawaiians who consumed the highest amount. Higher red meat intake mediated differences in *Lachnospira* (−41%) and *Ruminococcus-1* (36%) in Native Hawaiians over African Americans, who consumed the least. Dairy and alcohol intakes appeared to mediate and counterbalance the difference in *Bifidobacterium* between Whites and Japanese Americans. Conclusions: Overall diet quality and component food intakes may contribute to ethnic differences in GM composition and to GM-related racial/ethnic health disparities.

## 1. Introduction 

The human gut microbiome and its alterations have been associated with a wide array of metabolic and neuro-behavioral disorders, with especially strong evidence for obesity, inflammatory bowel disease, and colorectal cancer [1,2,3]. The majority of gut microbiome-disease associations are based on observational research, which limits causal inferences due to confounding and study heterogeneity [4]. Nevertheless, the consistency in observational findings across rigorously conducted studies [5] and the growing body of experimental evidence [6,7,8] support causal and sizable effects of the gut microbiota on a number of host conditions and translational potential for preventive and therapeutic applications [4,9]. 

A key feature of the human gut microbiome is the remarkable stability of its compositional structure within an individual over time while exhibiting large variability between individuals [6,10]. Conversely, it has also been suggested that even small proportional changes in gut microbial composition and functions may have a meaningful effect on host metabolism and health [11]. Research to date points to diet, body mass index (BMI), and aging as important determinants of such changes in healthy men and women [12,13]; however, little attention has been paid to racial/ethnic differences. Individual studies have either been based on mostly European Whites or limited in sample size for racial/ethnic comparative analyses [12], whereas pooled analyses of multiple studies for racial/ethnic comparisons are likely subject to confounding by geographic and cultural differences as well as technical analytic variation [14,15].

Two recent studies provide important initial evidence for gut microbial variation by race/ethnicity. Based on the 16S rRNA gene sequenced for microbial composition data from 1673 participants in two U.S. studies, Brooks et al. reported that the abundance of 12 microbial genera and families reproducibly varied by race/ethnicity across African Americans, Asians/Pacific Islanders, Hispanics, and Whites [16]. The race/ethnicity-distinguishing taxa included *Christensenellaceae*, known to be highly heritable [6], and others that have been associated with human genetic variation. Another study in the Netherlands reported that ethnicity (African, Middle Eastern, or South-Asian immigrants vs. Dutch) among the 2084 participants explained 5.7% of the dissimilarities in gut microbial composition [17]. These studies emphasized a critical need to further explore and account for racial/ethnic microbial differences in research, especially as an underlying mechanism for health disparities [18]. Although most of the racial/ethnic microbial variation may be random and benign to host health, certain variations have been systematically associated with social, economic, and structural stressors known to contribute to racial/ethnic health inequities [18].

While the aforementioned studies of the gut microbiome by race/ethnicity adjusted for limited dietary information to demonstrate persistent residual differences, the very effect of racially/ethnically varying dietary intake itself on gut microbial composition is of interest and merits investigation. In the current study, we assessed whether the racial/ethnic variation in gut microbial composition is mediated by dietary intake differences in a population-based prospective study of five race/ethnic groups with detailed dietary information and large 16S rRNA sequence-based fecal microbiome data. 

## 2. Methods

### 2.1. Study Population

Data from the Adiposity Phenotype Study (APS) [19] and microbiome genome-wide association study (mGWAS) [20] were combined for the current analysis. Both studies were conducted from 2013 to 2016 among a subset of the Multiethnic Cohort Study (MEC) participants, following a uniform protocol for stool sample collection and fecal microbiome analysis. The MEC is a population-based cohort of >215,000 men and women of five race/ethnic groups (African American, Japanese American, Latino, Native Hawaiian, and White) who were aged between 45 and 75 years and residing in Hawaii or Los Angeles County, California, at the baseline (1993–1996) [21]. Self-reported race/ethnicity was used for the cohort design and current analysis since self-identified race/ethnicity as a social construct is thought to capture different lived experiences and related individual behaviors and contextual factors [22]: in the MEC, self-reported race/ethnicity has been observed to be largely concordant with genetic ancestry [23,24].

For APS, a subset of 1861 MEC participants aged 60–77 years were re-recruited at the University of Hawaii (UH) and the University of Southern California (USC) for racial/ethnic comparisons of body fat distribution [19]. Participants were enrolled stratified on sex, race/ethnicity, and six BMI categories (18.5–21.9, 22–24.9, 25–26.9, 27–29.9, 30–34.9, and 35–40 kg/m^2^ excluding reported BMIs outside this range) in order to facilitate comparisons across sex-ethnic groups. For mGWAS, a non-overlapping subset of MEC participants with prior GWAS data was re-contacted to collect a stool sample via mailing (*n =* 4502; aged 60–90 years) for a study of human genetics associated with the gut microbiome [20]. The overall participation rate was 23% for the APS and 43% for the mGWAS among the invited and eligible individuals. The Institutional Review Boards at UH and USC approved the study protocols, and all participants provided informed consent, adhering to the principles in the Belmont Report [25].

For both APS and mGWAS, individuals were excluded if they had a history of ileostomy or colectomy or if they were affected by advanced diseases such as dialysis. For the APS that involved body imaging, willing MEC participants were additionally excluded for recent smoking in the past two years and contraindications for magnetic resonance imaging (MRI) or dual energy X-ray absorptiometry (DXA) such as claustrophobia, soft or metal body implants, and amputation. Participation in both APS and mGWAS was deferred if participants reported the following conditions that could affect systemic metabolism or the gut microbiota [19,20]: recent treatment with chemotherapy, radiation therapy, corticosteroid hormones, prescription weight-loss drugs, insulin or thyroid medications (<6 months); recent endoscopy, irrigation or cleansing of the large intestine (<6 months); recent weight change over 20 lbs (or 9.072 kg; <6 months); recent antibiotics (<3 months); or recent flu shot or other vaccination (<1 month). Participation in APS was also deferred for recent imaging with contrast (<2 months). 

### 2.2. Stool Collection and Fecal Microbiome Analysis

Participants were provided with a stool collection kit and instructions to collect a sample at home into a vial containing RNAlater, store the sample overnight in their home freezer, and return it with responses to a short questionnaire such as antibiotic use in the past year (yes/no) [20]. The received samples were stored at −80 °C until shipping on dry ice to the Fred Hutchinson Cancer Research Center (Fred Hutch) for analysis.

The protocols for laboratory analysis and bioinformatic data processing have been described in detail [10,26] and also included in the online Supplementary Materials Figures S1 and S2), along with information on data sharing. Briefly, DNA from stool samples was extracted and amplified for the V1–V3 region of the 16S rRNA gene at Fred Hutch and shipped to the Research and Testing Laboratory (RTL LLC, Lubbock, TX, USA) for sequencing. Fecal microbial composition was assessed with 2 × 300 bp paired-end sequencing on the Illumina MiSeq platform. Quality control of sequences and taxa identification were conducted using the Quantitative Insights Into Microbial Ecology (QIIME) v1.8 pipeline implementing the SILVA v132 reference database [27]. 

### 2.3. Dietary Assessment

Dietary data from the 10-year follow-up in the MEC (2003–2008) were used in the current analysis since they were the most recent and commonly available assessment prior to the date of stool collection. Dietary intake was assessed with a self-administered, quantitative food frequency questionnaire (QFFQ) that was developed and validated specifically for the MEC study population [21,28]. The MEC QFFQ included questions related to usual eating habits for over 180 food or food groups during the past year. For each food item, participants were asked to select one of eight consumption frequency categories and one of three portion sizes for a typical serving. Overall diet quality of each participant was scored based on their adherence to dietary recommendations by the Healthy Eating Index (HEI)-2015): HEI scoring is based on absolute intake amounts, and thus is more comparable over time and across populations compared to other indices [29]. To additionally examine specific food groups, we analyzed the intake amounts of 14 individual food groups (or nutrients) that are used to define any of the four indices (HEI-2015, the Alternative Healthy Eating Index, the alternate Mediterranean Diet and the Dietary Approaches to Stop Hypertension) [30]. The individual food/nutrient groups included seven ‘adequacy’ components (fruits, vegetables, nuts/seeds/legumes, whole grains, dairy, fish, monounsaturated to saturated fatty acid ratio (MUFA/SFA)) and seven ‘moderation’ components (alcohol, red meat, refined grains, added sugars, sugar-sweetened beverages, saturated fat, sodium). 

### 2.4. Statistical Analysis

Of the 6363 participants in total in the APS and mGWAS, gut microbiome data were available for 6094 (see Supplementary Figure S3 for the participant flow chart). We excluded participants with invalid diet data (e.g., implausible values for total energy and component macronutrients [31]; *n =* 149) or missing BMI values (*n =* 9) at cohort entry. We further excluded those with missing or invalid diet data at the 10-year follow-up (*n =* 656) and those in mGWAS with missing BMI (*n =* 13), retaining 5267 participants for the current analysis. 

Before the mediation analysis, we examined mean dietary intake patterns (HEI-2015 total score for overall diet quality and intake amounts of 14 component food groups or nutrients) in general linear models (SAS, v9.4; PROC GLM), adjusted for age, sex, energy intake, and BMI, and compared them across race/ethnic groups. All dietary variables, except HEI-2015, were log-transformed to meet the model assumptions. 

The association between gut microbial composition and race/ethnicity was similarly examined in general linear models. Gut microbial composition was analyzed as the relative abundance of genera, as determined by the centered log-ratio (CLR) transformed count of each genus (R, v4.1.2). Of the 152 genera, we focused on 63 common genera that were present in at least 50% of the participants in all five race/ethnic groups, consistent with an approach in a previous multiethnic comparison [16]. 

We performed mediation analysis to determine whether the effect of race/ethnicity on gut microbial composition is in part mediated by overall diet quality or component food/nutrient intake (Supplementary Figure S4). We used causal mediation analysis based on the counterfactual-based framework [32], specifically using the marginal structural models to impute the counterfactuals [33]. Total effect of race/ethnicity—a multi-categorical exposure (five race/ethnic groups) [34]—on the abundance of each genus was partitioned into indirect effects mediated through each of the dietary factors and direct effects (remaining racial/ethnic differences in the genus driven by other factors), while adjusting for potential confounding by age, sex, energy intake, BMI, and antibiotic use. Using the R package medflex [35], counterfactuals were imputed based on the food mediator (neImpute), and a natural effect model (neModel) was fit using the linear regression of each genus (CLR value) on the observed and the counterfactual race/ethnicity and covariates to estimate their regression coefficients and standard errors for total, direct, and indirect effects from 100 bootstrap replications. For each dietary factor, the race/ethnic group with the most desirable mean intake level was used as the reference (i.e., the group with highest mean HEI-2015 score, highest mean intake of ‘adequacy’ components such as vegetables, or lowest mean intake of ‘moderation’ components such as red meat). A mediation (or indirect) effect with *p <* 1.3 × 10^−5^ was selected as significant based on Bonferroni correction for 945 mediation models on 63 genera and 15 dietary factors, with comparisons of four race/ethnic groups to the reference group in each model. For each significant mediation/indirect effect, the proportion (%) mediated by the given dietary factor was determined as the ratio of regression coefficients for the indirect over the total race/ethnicity effect. 

Finally, the genera with significant mediation by one or more dietary factors were examined in mean abundance, adjusted for age, sex, BMI, and antibiotic use across the race/ethnic groups using the general linear model (and median regression for p-value; SAS, PROC QUANTREG). The Spearman correlation between the genera and dietary factors was examined in a heatmap (R package heatmap.2).

## 3. Results

The study population was comprised of similar numbers of men and women and a large proportion (>82%) of individuals from minority groups (Table 1). The distribution of the sex-ethnic subgroups reflects that in the parent MEC and its GWAS subset including more Japanese Americans than other groups and more women than men among African Americans. The mean age of the participants was 65.3 years at dietary assessment (2003–2008) and 74.6 years at stool collection (2013–2016), with an interval of 9.3 (±1.4) years. Both mean BMI and the prevalence of weight categories varied substantially by race/ethnicity. Some variations were also observed in the proportion of current smokers, mean energy intake, and the recent history of antibiotic use. 

Table 2 illustrates the large racial/ethnic differences in overall diet quality (HEI-2015) and intake amounts of 14 component foods/nutrients. After adjustment for age, sex, energy intake and BMI, the mean overall diet quality was highest in African Americans (72.9) and Whites (72.6), followed by Japanese Americans (70.4) and Native Hawaiians (70.2), and lowest in Latinos (68.9) (*p <* 0.0001). This was consistent with the pattern observed in the parent MEC, where the mean adjusted HEI-2015 score was highest in Whites (69.2; 95% confidence limit: 69.1–69.3) and African Americans (69.1; 69.0–69.2) and lowest in Latinos (65.7; 65.6–65.7) (data not shown). For each dietary factor, the race/ethnic group with the most desirable mean intake level is noted in bold in Table 2.

**Figure 1:**
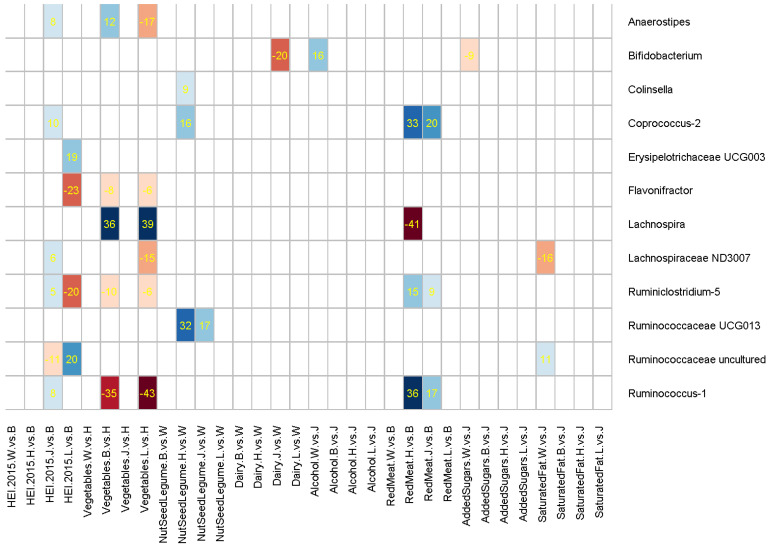
Mediating effects of dietary factors for the racial/ethnic differences in 12 gut microbial genera. The heatmap cells are shaded for positive (blue) or negative (red) mediation effects of statistical significance (*p <* 1.32 × 10^−5^ for Bonferroni correction), with numbers inserted for the percent of total ethnicity effect mediated by each dietary variable.

Supplementary Table S1 describes the gut microbiome in terms of alpha diversity and relative abundance of the phyla among the study participants, overall and by race/ethnicity. We then examined alpha diversity indices and relative abundance of the 12 phyla and 63 common genera by race/ethnicity and decomposed the racial/ethnic differences into indirect effects mediated through dietary factors and other direct effects. Supplementary Table S2 shows the total, direct, and indirect effects of race/ethnicity on the 12 genera, for which one or more of the dietary factors had significant mediation: after adjusting for multiple tests, dietary mediation was not significant for alpha diversity indices, 12 phyla, or 51 common genera. Figure 1 illustrates the significant positive (blue) or negative (red) mediation by one or more of eight dietary factors for the race/ethnicity effect on the abundance of 12 bacterial genera. The figure does not show the other 51 genera or seven dietary factors (fruits, whole grains, fish, MUFA/SFA ratio, refined grains, sugar-added beverages, and sodium) that did not involve significant racial/ethnic differences or significant dietary mediation. Positive mediation by diet is likely to have contributed to the overall ethnic difference observed in the genus abundance, whereas negative mediation is likely to have reduced otherwise a larger ethnic difference in the genus caused by the direct effect of other factors. 

Overall diet quality (HEI-2015) and intakes of vegetables and red meat mediated the gut microbiome differences across ethnic groups more frequently than others (Figure 1). HEI-2015 most notably mediated the ethnic differences in *Erysipelotrichaceae UCG003* (19%), *Flavonifractor* (−23%), *Ruminiclostridium-5* (−20%), and *Ruminococcaceae uncultured* (20%) between African Americans and Latinos, groups with the highest and lowest mean HEI-2015, respectively. Compared to Native Hawaiians, lower vegetable intake among African Americans and Latinos mediated their relative abundance of *Lachnospira* (36% for African Americans, 39% for Latinos) and *Ruminococcus-1* (−35%, −43%). Red meats, consumed the most by Native Hawaiians and the least by African Americans, mediated the differences in *Coprococcus-2* (33%), *Lachnospira* (−41%), and *Ruminococcus-1* (36%). 

In addition, the intake of nuts, seeds, or legumes mediated the differences in *Ruminococcaceae UCG013* to the largest degree (32%) among Native Hawaiians compared to Whites, groups with the lowest and highest mean intake levels, respectively. The abundance of *Bifidobacterium* appeared to be enhanced by dairy intake and reduced by alcohol intake. Compared to Whites with the highest consumption of both dairy and alcohol, Japanese Americans had a higher abundance of *Bifidobacterium* (total effect coefficient = 0.4960, Supplementary Table S2), which was positively mediated by their lower alcohol intake (16%) and which would have been by a larger margin if not for the negative mediation by their lower dairy intake (−20%).

When overall diet quality was adjusted for in the models for component foods/nutrients in order to assess independent associations of the latter, the mediation by individual foods/nutrients was generally attenuated in magnitude and significance (Supplementary Table S3). One exception was the difference in *Ruminococcus-1* between African Americans and Native Hawaiians mediated by vegetable intake, which was strengthened from −35% to −46%. 

The 12 genera in Figure 1 with significant mediation by dietary factors are shown in Supplementary Table S4 for mean relative abundance across race/ethnic groups, adjusted for age, sex, BMI, and antibiotic use. Supplementary Figure S5 shows unadjusted Spearman correlation coefficients for the 12 genera in relation to dietary factors and key covariates in a heatmap, showing generally weak associations and indicating that simple correlations are not likely to show the complexity of the inter-relationships.

## 4. Discussion 

In this large, ethnically diverse study population, we found significant racial/ethnic variation in gut microbial composition and in the contributions to these differences by some of the dietary intake patterns considered. Among the 63 genera that were prevalent in all five of our racial/ethnic populations, the ethnic difference in 12 genera was significantly mediated by eight of the 15 key dietary factors examined. The absolute magnitude of the mediation effects, as determined by the percent of total ethnicity effect on the given genus abundance mediated by a dietary factor, was as high as 43%. Notably, strong dietary mediation (absolute percent mediation ≥30%) was detected for *Coprococcus-2, Lachnospira, Ruminococcaceae UCG013*, and *Ruminococcus-1* by racial/ethnic differences in the dietary consumption of vegetables, nuts/seeds/legumes, or red meat.

In this analysis, we primarily focused on the dietary mediation of the ethnic variation. We observed that about 40% of 152 genera were common across the five race/ethnic groups in our population. The common genera included four of the 12 taxa that differentiated race/ethnicity well in the only prior U.S. study comparing gut microbial composition across 88 Asian-Pacific Islanders, 1237 Caucasians, 37 Hispanics, and 13 African Americans [16]: *Christensenellaceae, Odoribacter, Alistipes,* and *Collinsella*. *Christensenellaceae* and *Alistipes* ranked high in their racial/ethnic differences in our data. An additional three (*Veillonella, Verrucomicrobiaceae*, and *Victivallaceae*) of the 12 were found at low prevalence in our data, and the other five were not detected in quantifiable amounts (data not shown). Although reasons for this discrepancy between the previous study vs. ours are not clear, some may be due to the differences in the 16S rRNA gene region sequenced, the reference database, and collection/storage conditions as well as the limited sample size of minority individuals. We have shown that the stool collection protocol used in our analysis had high reliability in detecting abundant taxa [10]. Regardless, we need additional studies that are inclusive of racial/ethnic minorities, rigorous in collecting high quality data, and specific whenever possible to disaggregate racial/ethnic (sub)groups with potentially different microecology such as Americans of Asian vs. Pacific Islander descent. 

We found that the magnitude of dietary mediation was not proportional to the ethnic difference in individual genera, indicating varying effects of non-dietary determinants on the gut microbiome. For example, *Christensenellaceae*, *Blautia*, and *[Eubacterium] ventriosum* ranked high in their importance to differentiate race/ethnic groups (data not shown) but showed little mediation by dietary intake differences. Similarly, others found no association between diet and *Christensenellaceae* [36]. 

Overall diet quality had the largest mediation effect on the ethnic variation in the abundance of *Flavonifractor*, *Ruminiclostridium-5*, and *Ruminococcaceae uncultured*. *Flavonifractor* is known to catabolize catechin flavonoids in the gut [37]. *Flavonifractor plautii* has specifically been associated with a high-fat/low-fiber diet [38], increased gut permeability [39], and colorectal cancer [40], while another taxon, *Flavonifractor OTU41*, was reduced in a randomized trial of cranberry supplementation rich in polyphenols [41]. *Ruminiclostridium-5* can metabolize complex polymers such as cellulose, xylan, and N-acetylglucosamine as an energy source [42] and was increased in a randomized trial of a polydextrose fiber supplement [43]. Thus, our finding is supported by the known dependence of these genera on host diet.

We also found that the racial/ethnic differences in the abundance of *Lachnospira* and *Ruminococcus-1* were mediated substantially and in opposite directions by the intake of vegetables and red meat. *Lachnospira*, a member of short-chain fatty acid (SCFA) producer *Lachnospiraceae*, has been inversely associated with weight gain and obesity [44] and was increased in studies that supplemented dietary fiber or vitamin D [45]. Enriched *Ruminococcus* species within the phylum *Firmicutes* have been linked to inflammatory bowel diseases [46] and was reduced after a probiotic intervention [47]. Consistent with the favorable profile of *Lachnospira* and the deleterious profile of *Ruminococcus-1*, we observed that African Americans and Latinos, groups with low vegetable intake, had lower abundance of *Lachnospira* and higher abundance of *Ruminococcus-1* compared with Native Hawaiians who reported the highest vegetable intake; 35–43% of these differences was mediated by the vegetable intake difference. In contrast, red meat intake, reported to be higher in Native Hawaiians than African Americans, showed a counter-balancing mediation effect for the ethnic difference in *Lachnospira* and *Ruminococcus-1* abundance.

The difference in *Bifidobacterium* between Whites and Japanese Americans was mediated by their dairy and alcohol intake [48]. Whites reported the highest mean dairy intake but had the lowest abundance of *Bifidobacterium*, and the genus difference compared to Japanese Americans was mediated in part (16%) by higher alcohol intake among Whites, indicating that alcohol had a suppressing effect on the genus [49]. It is likely that Japanese Americans could have had a greater abundance in *Bifidobacterium* over Whites had they increased their low intake of fermented dairy foods (−20%). *Bifidobacterium* members are widely commercialized as probiotics, known for their SCFA-producing and beneficial immunomodulatory properties [50]. While *Bifidobacterium* abundance has been consistently linked to human genetic variation such as for lactase persistence (lower in Asian Americans than in Whites), it has been suggested that the influence of environment prevails over host genetics [51]. Our findings similarly underscore the importance of dietary exposures and further allude to their potential counter-acting effects of different dietary components.

There are several important strengths to this study. A large number of diverse participants were included. Stool samples were collected into a nucleic acid fixative, which has been shown to provide high-quality and reproducible results comparable to that obtained from fresh-frozen collection protocols [52,53]. Dietary assessment was based on a QFFQ instrument specifically developed and validated for a multiethnic population [28]. Racial/ethnic comparisons were made with the group with the most desirable level of each dietary factor as the reference, which allowed for the detection of maximum dietary mediation compared to having Whites as the common reference.

On the other hand, there were some limitations in our analyses. We analyzed the dietary intake data from 10 years prior to the stool collections, which may have yielded different associations than a more recent diet. We had information on concurrent diet only for the APS subset (*n =* 1484), where similar mediation trends were observed but did not reach statistical significance after adjustment for multiple tests (Supplementary Table S5), likely due to the smaller sample size. While transient changes in gut microbial composition have been observed in response to dietary fluctuations such as fiber intake [54], the overall long-term stability of adult gut microbiome is well established [55,56], supporting a relatively consistent association with habitual diet over time. Although direct studies are nearly nonexistent for associations with habitual diet in the distant past [57], our longitudinal analysis showed that dietary intake patterns up to 20 years prior to stool collection had a consistent association as concurrent dietary intake with measures of fecal microbial community structure [58], and we expect to see more longitudinal analyses from other similar cohorts. Additionally, the race/ethnic groups in the MEC are mostly separated geographically, with African Americans and Latinos mostly from California and Japanese Americans, Native Hawaiians and Whites mostly from Hawaii, which limited our ability to differentiate racial/ethnic variation from geographic variation in the gut microbiome. We observed some evidence, however, that the geographic variation may not be substantial and may be limited to a subgroup of the microbial genera in our comparison of small subsets of Japanese Americans and Whites from California with their counterparts from Hawaii ( Supplementary Table S6). In addition, although we adjusted for key correlates, other determinants might have confounded some of the mediation effects. Finally, considering the functional redundancy of the human microbiome [59], our compositional analysis might have overestimated the ethnic differences or their mediation by dietary intake: future studies with high-resolution taxonomic and metagenomic analysis are warranted to more accurately characterize the racial/ethnic variation and the dietary mediation. 

In conclusion, we made a novel observation of substantial dietary mediation for the significant racial/ethnic variation in gut microbial composition. This is an important finding to understand the biological underpinning of racial/ethnic health disparities and to identify relevant targetable dietary interventions.

## Figures and Tables

**Table 1:** Characteristics * of study participants.

	Overall	White	African American	Native Hawaiian	Japanese American	Latino
N	5267	918	750	684	1969	946
Sex, % women	51%	49%	60%	57%	49%	48%
Age at dietary assessment, mean (SD)	65.3 (6.9)	64.3 (6.6)	65.8 (7.3)	63.3 (6.5)	65.9 (7.1)	66.0 (6.3)
Age at stool collection, mean (SD)	74.6 (6.9)	73.5 (6.6)	75.3 (7.2)	72.8 (6.6)	75.1 (7.1)	75.6 (6.4)
BMI, mean kg/m^2^(SD)	26.4 (5.1)	26.0 (4.9)	28.3 (5.5)	28.3 (5.5)	24.8 (4.4)	27.7 (5.0)
Normal-weight, %	43%	45%	30%	31%	57%	31%
Overweight, %	37%	37%	40%	36%	34%	42%
Obese, %	20%	18%	31%	33%	10%	27%
Smoking status, %						
Never	47%	46%	43%	42%	51%	47%
Former	42%	41%	47%	46%	40%	41%
Current	9%	12%	12%	10%	9%	12%
Energy intake, mean kcal/day (SD)	1869 (810)	1925 (712)	1660 (838)	2067 (997)	1789 (653)	2006 (952)
Antibiotics use in the past year, %	19%	21%	17%	17%	17%	21%

* The characteristics are from the 10-year follow-up of the Multiethnic Cohort (2003–2008; age at dietary assessment, BMI, energy intake and smoking status) or from the Adiposity Phenotype Study and microbiome GWAS (2013–2016; age at stool collection, antibiotics use). The characteristics are presented in percent for categorical traits and mean (standard deviation (SD)) for continuous traits.

**Table 2:** Mean (95% confidence limit) overall diet quality and component food/nutrient intake by race/ethnicity.

Dietary Factors	White (*n =* 918)	African American (*n =* 750)	Native Hawaiian (*n =* 684)	Japanese American (*n* = 1969)	Latino (*n =* 946)	*p*
Overall diet quality (HEI-2015)	72.6 (71.1, 74.0)	**72.9 (71.4, 74.4)**	70.2 (68.6, 71.7)	70.4 (69.0, 71.8)	68.9 (67.5, 70.4)	1.5 × 10^−19^
Fruits (cups/day)	1.94 (1.71, 2.18)	1.94 (1.71, 2.18)	1.99 (1.75, 2.23)	1.71 (1.49, 1.93)	**2.30 (2.08, 2.53)**	3.1 × 10^−17^
Vegetables (cups/day)	2.32 (2.11, 2.52)	1.84 (1.63, 2.05)	**2.51 (2.30, 2.72)**	2.19 (1.99, 2.38)	2.04 (1.84, 2.24)	1.2 × 10^−19^
Nuts, seeds, legumes (g/day)	**37.4 (32.6, 42.0)**	30.1 (25.2, 34.9)	30.9 (26.1, 35.7)	30.9 (26.4, 35.4)	29.5 (24.7, 34.0)	9.0 × 10^−7^
Whole grains (g/day)	**52.4 (46.8, 58.1)**	51.3 (45.6, 57.0)	52.4 (46.5, 58.1)	48.2 (42.8, 53.6)	42.0 (36.6, 47.6)	5.9 × 10^−9^
Dairy (cups/day)	**1.54 (1.42, 1.67)**	1.00 (0.87, 1.12)	1.15 (1.02, 1.28)	0.88 (0.77, 1.00)	1.53 (1.41, 1.65)	2.5 × 10^−114^
Fish (g/day)	26.4 (23.0, 30.1)	23.2 (19.6, 26.9)	**35.7 (32.0, 39.4)**	31.2 (27.8, 34.6)	18.7 (15.3, 22.1)	1.6 × 10^−54^
MUFA/SFA ratio	1.21 (1.18, 1.24)	1.29 (1.26, 1.32)	1.28 (1.25, 1.32)	**1.36 (1.33, 1.39)**	1.23 (1.20, 1.26)	5.0 × 10^−86^
Alcohol (g/day)	12.9 (10.8, 15.0)	6.7 (4.6, 8.8)	7.5 (5.3, 9.7)	**4.1 (2.2, 6.1)**	6.4 (4.4, 8.4)	9.0 × 10^−47^
Red meat (g/day)	42.8 (38.0, 47.6)	**32.9 (28.1, 37.7)**	55.0 (50.2, 59.8)	47.9 (43.4, 52.4)	40.8 (36.3, 45.6)	1.7 × 10^−40^
Refined grains (g/day)	105 (94, 115)	**90 (80, 101)**	141 (130, 152)	139 (129, 150)	151 (140, 161)	4.1 × 10^−87^
Added sugars (tsp/day)	9.55 (8.68, 10.4)	9.38 (8.50, 10.2)	9.74 (8.85, 10.6)	**7.38 (6.56, 8.19)**	9.43 (8.59, 10.3)	1.1 × 10^−30^
Sugar-sweetened beverages (g/day)	90 (64, 116)	145 (119, 172)	117 (90, 144)	**80.2 (56, 105)**	120 (95, 145)	8.2 × 10^−17^
Saturated fat (g/day)	23.1 (21.6, 24.6)	19.7 (18.2, 21.2)	23.6 (22.1, 25.2)	**19.2 (17.7, 20.6)**	23.7 (22.2, 25.1)	1.6 × 10^−42^
Sodium (g/day)	3.15 (2.94, 3.35)	**2.66 (2.46, 2.87)**	3.47 (3.26, 3.68)	3.12 (2.93, 3.32)	3.34 (3.15, 3.54)	5.2 × 10^−29^

The mean (95% confidence limit) for the overall diet quality (the Healthy Eating Index (HEI-2015) score) and the intake amounts of diet quality-defining component foods/nutrients was obtained in a general linear model of each dietary factor on race/ethnicity, adjusted for age, sex, BMI and energy intake, along with the p-value for ethnic differences. Measurement equivalents: 1 cup is equivalent to 236.588 mL; 1 teaspoon is equivalent to 4.92892 mL. For each dietary factor, the race/ethnic group with the most desirable (the most of the adequacy item or the least of the moderation item) mean intake level is noted in bold.

## Data Availability

Data described in the manuscript, code book, and analytic code will be made available upon request. In addition, all of the gut microbiome sequencing data will be submitted to the Sequence Read Archive (https://www.ncbi.nlm.nih.gov/sra, accessed on 10 December 2021) with a project code added to the paper once accepted for publication.

## Supplementary Materials

The following supporting information can be downloaded at: https://www.mdpi.com/article/10.3390/nu14030660/s1, including supplemental method description [60,61,62,63,64,65,66,67,68,69,70,71,72]; Figure S1: Flow chart for the gut microbiome data processing; Figure S2: Dependency graph showing the links between processing steps, source files, and output files for the Multiethnic Cohort Study 16S datafiles after OTU generation; Figure S3: Participant flow chart; Figure S4: Conceptual framework for the current mediation analysis; Figure S5: Spearman correlations between 15 dietary factors and 12 gut microbial genera that showed significant mediation; Table S1: Description (mean (standard deviation)) of gut microbial diversity and phyla abundance, overall and by race/ethnicity; Table S2: Decomposition of total ethnicity effect on 63 common genera abundance into direct effects and indirect effects mediated through dietary factors, adjusted for age, sex, energy intake, BMI and antibiotics use; Table S3: Decomposition of total ethnicity effect on gut microbial genera abundance into direct effects and indirect effects mediated through dietary factors, adjusted for overall diet quality (HEI-2015), as well as age, sex, energy intake, BMI and antibiotics use; Table S4: Mean relative abundance of 12 genera most mediated by dietary factors, stratified by race/ethnicity; Table S5: Decomposition of total ethnicity effect on the 12 genera abundance from the main analysis (Table S1) into direct effects and indirect effects mediated through dietary factors using the concurrent Food Frequency Questionnaire data available in the Adiposity Phenotype Study subset (n=1,484), adjusted for age, sex, energy intake, BMI and antibiotics use; Table S6: Comparison of 15 dietary factors and 12 genera by study area within the same race/ethnicity of Whites or Japanese Americans.

## Abbreviations

APS: Adiposity Phenotype Study; HEI, the Healthy Eating Index; MEC, the Multiethnic Cohort Study; mGWAS, microbiome Genome-Wide Association Study; QFFQ, quantitative food frequency questionnaire.
