# Supplementary material for: Dietary Intake Mediates Ethnic Differences in Gut Microbial Composition
Source: Nutrients. 2022 Feb 4;14(3):660. doi: 10.3390/nu14030660 (PMC8840192; doi:10.3390/nu14030660)
Supplement: Supplementary file 1 [file nutrients-14-00660-s001.zip › nutrients-1527306-supplementary.pdf]

ONLINE SUPPLEMENTAL MATERIAL

Methods

2.2 Stool collection and fecal microbiome analysis – quality control and data processing

The protocols for laboratory analysis and bioinformatic data processing have been described in great detail [1,2] and also summarized below: see the flow chart in **Supplementary Figure S1 and Supplementary Figure S2**. Briefly, DNA from stool samples was extracted and amplified for the V1-V3 region of the 16S rRNA gene at Fred Hutch and shipped to Research and Testing Laboratory (RTL LLC, Lubbock, TX) for sequencing. Fecal microbial composition was assessed with 2x300 bp paired-end sequencing on the Illumina MiSeq platform. Quality control of sequences and taxa identification were done using the Quantitative Insights Into Microbial Ecology (QIIME) v1.8 pipeline implementing the SILVA v132 reference database [3].

Filtering of operational taxonomic units (OTUs): The filtering strategy for OTUs included using parameters in QIIME to exclude low abundance sequences, singletons, and chimeras. Briefly, the OTU table was filtered using QIIME script filter_OTUs_from_OTU_table.py with -min_count_fraction set to 0.00005. Additional OTU entries were filtered out if they were detected as chimeras using QIIME's identify_chimeric_seqs.py script with method blast_fragments. Final filtering excluded genera that appeared in <10% of the participants [4].

Taxonomic identification/classification against reference library: The QIIME-processed sequences were aligned to the SILVA v132 database (release 111) as the reference library for 16S rRNA gene classification [5] using the PyNAST algorithm [6]. Sequences were joined with the fastq-join method, using min_overlap=15 and perc_max_diff=12. After filtering sequences, as described above, the Nelson two-step method was used for OTU generation at 97% similarity with the SILVA database for closed reference OTU picking following the UCLUST algorithm [7]. Specifically, the sequences were classified using the matching SILVA taxonomy for OTUs found in the first step of the Nelson method, and using MOTHUR's naïve Bayesian Classifier [8,9] trained against the SILVA database for OTUs found in the second step. Sequences that did not align to the appropriate 16S rRNA gene region were removed.

Sequence counts in each sample for the phylum and genus level were generated without rarefaction. Sequence reads ranged at 9,831-178,452 (mean=38,029, SD=19,034; median=34,008). The phylogenetic tree was constructed following the FastTree method [10]. Alpha diversity measures (phylogenetic diversity [11]; Shannon index [12]; Chao1 index [13]) and beta diversity matrices (unweighted and weighted UniFrac [14,15]) were calculated in QIIME based on the average of 10 subsamples with rarefaction to 10,000 sequences per sample.

Quality control: We included duplicate participant samples, blind quality control (QC) samples, and blanks to assess variation in library preparation and sequencing batches. Bacterial DNA concentration and purity were determined using the NanoDrop 8000 Spectrophotometer (Thermo Fisher Scientific) and gel electrophoresis, from which two aliquots from each participant at a final concentration of 20 ng/uL were processed together in the same batch for sequencing. Fred Hutch control (FHC) samples were prepared as blind QCs and inserted across sequencing batches [1]. Intraclass correlation coefficients (ICC) for extraction duplicates were ≥0.93 for alpha

diversity measures, ≥0.99 for the first PCoA axis for unweighted and weighted UniFrac, and ≥0.97 for the four most abundant phyla [1].

Batch adjustment and normalization: We processed the >6,000 fecal DNA samples in 32 batches that were analyzed in 4 groups over the study period: batches 1-9 in group A; batches 10-13 in group B; batches 14-20 in group C; and batches 22-32 in group D. We used R package combat to adjust for any differences in bacterial frequency data in duplicates and QCs across the groups and batches. We then performed the centered log-ratio transformation to normalize the combat-adjusted abundance data.

Data sharing: We will post the gut microbiome sequence data on the Sequence Read Archive database (https://www.ncbi.nlm.nih.gov/sra). We will also make available the processed gut microbiome genera data with key covariates to any inquiries following the Resource Sharing Policy established in the Multiethnic Cohort Study (https://www.uhcancercenter.org/pdf/mec/MEC%20Data%20Sharing%20Policy_09-02-20.pdf), which involves submitting an online application (https://www.uhcancercenter.org/for-researchers/mec-data-sharing).

REFERENCES for Supplemental Material

1. Fu, B.C.; Randolph, T.W.; Lim, U.; Monroe, K.R.; Cheng, I.; Wilkens, L.R.; Le Marchand, L.; Lampe, J.W.; Hullar, M.A.J. Temporal Variability and Stability of the Fecal Microbiome: The Multiethnic Cohort Study. *Cancer Epidemiol Biomarkers Prev* **2019**, *28*, 154-162, doi:10.1158/1055-9965.EPI-18-0348.
2. Hullar, M.A.J.; Jenkins, I.C.; Randolph, T.W.; Curtis, K.R.; Monroe, K.R.; Ernst, T.; Shepherd, J.A.; Stram, D.O.; Cheng, I.; Kristal, B.S., et al. Associations of the gut microbiome with hepatic adiposity in the Multiethnic Cohort Adiposity Phenotype Study. *Gut Microbes* **2021**, *13*, 1965463, doi:10.1080/19490976.2021.1965463.
3. Quast, C.; Pruesse, E.; Yilmaz, P.; Gerken, J.; Schweer, T.; Yarza, P.; Peplies, J.; Glockner, F.O. The SILVA ribosomal RNA gene database project: improved data processing and web-based tools. *Nucleic Acids Res* **2013**, *41*, D590-596, doi:10.1093/nar/gks1219.
4. Langille, M.G.I.; Zaneveld, J.; Caporaso, J.G.; McDonald, D.; Knights, D.; Reyes, J.A.; Clemente, J.C.; Burkepile, D.E.; Thurber, R.L.V.; Knight, R., et al. Predictive functional profiling of microbial communities using 16S rRNA marker gene sequences. *Nat Biotechnol* **2013**, *31*, 814-821, doi:Doi 10.1038/Nbt.2676.
5. Pruesse, E.; Quast, C.; Knittel, K.; Fuchs, B.M.; Ludwig, W.; Peplies, J.; Glockner, F.O. SILVA: a comprehensive online resource for quality checked and aligned ribosomal RNA sequence data compatible with ARB. *Nucleic Acids Res* **2007**, *35*, 7188-7196, doi:10.1093/nar/gkm864.
6. Caporaso, J.G.; Bittinger, K.; Bushman, F.D.; DeSantis, T.Z.; Andersen, G.L.; Knight, R. PyNAST: a flexible tool for aligning sequences to a template alignment. *Bioinformatics* **2010**, *26*, 266-267, doi:10.1093/bioinformatics/btp636.
7. Edgar, R.C. Search and clustering orders of magnitude faster than BLAST. *Bioinformatics* **2010**, *26*, 2460-2461, doi:10.1093/bioinformatics/btq461.
8. Schloss, P.D.; Westcott, S.L.; Ryabin, T.; Hall, J.R.; Hartmann, M.; Hollister, E.B.; Lesniewski, R.A.; Oakley, B.B.; Parks, D.H.; Robinson, C.J., et al. Introducing mothur: open-source, platform-independent, community-supported software for describing and comparing microbial communities. *Applied and environmental microbiology* **2009**, *75*, 7537-7541, doi:10.1128/AEM.01541-09.
9. Wang, Q.; Garrity, G.M.; Tiedje, J.M.; Cole, J.R. Naive Bayesian classifier for rapid assignment of rRNA sequences into the new bacterial taxonomy. *Applied and environmental microbiology* **2007**, *73*, 5261-5267, doi:10.1128/AEM.00062-07.
10. Price, M.N.; Dehal, P.S.; Arkin, A.P. FastTree 2--approximately maximum-likelihood trees for large alignments. *PLoS One* **2010**, *5*, e9490, doi:10.1371/journal.pone.0009490.
11. Faith, D.P.; Baker, A.M. Phylogenetic diversity (PD) and biodiversity conservation: some bioinformatics challenges. *Evol Bioinform Online* **2007**, *2*, 121-128.
12. Shannon, C.E.; Weaver, W. *The mathematical theory of communication*; The University of Illinois Press: Illinois, 1998.
13. Chao, A.; Shen, T.J. Nonparametric estimation of Shannon's index of diversity when there are unseen species in sample. *Ecol Stat* **2003**, *10*, 429-443.
14. Lozupone, C.; Knight, R. UniFrac: a new phylogenetic method for comparing microbial communities. *Applied and environmental microbiology* **2005**, *71*, 8228-8235, doi:10.1128/AEM.71.12.8228-8235.2005.
15. Lozupone, C.A.; Hamady, M.; Kelley, S.T.; Knight, R. Quantitative and qualitative beta diversity measures lead to different insights into factors that structure microbial communities. *Applied and environmental microbiology* **2007**, *73*, 1576-1585, doi:10.1128/AEM.01996-06.
16. Nelson, M.C.; Morrison, H.G.; Benjamino, J.; Grim, S.L.; Graf, J. Analysis, optimization and verification of Illumina-generated 16S rRNA gene amplicon surveys. *PLoS One* **2014**, *9*, e94249, doi:10.1371/journal.pone.0094249.

Figure S1. Flow Chart for the gut microbiome data processing.

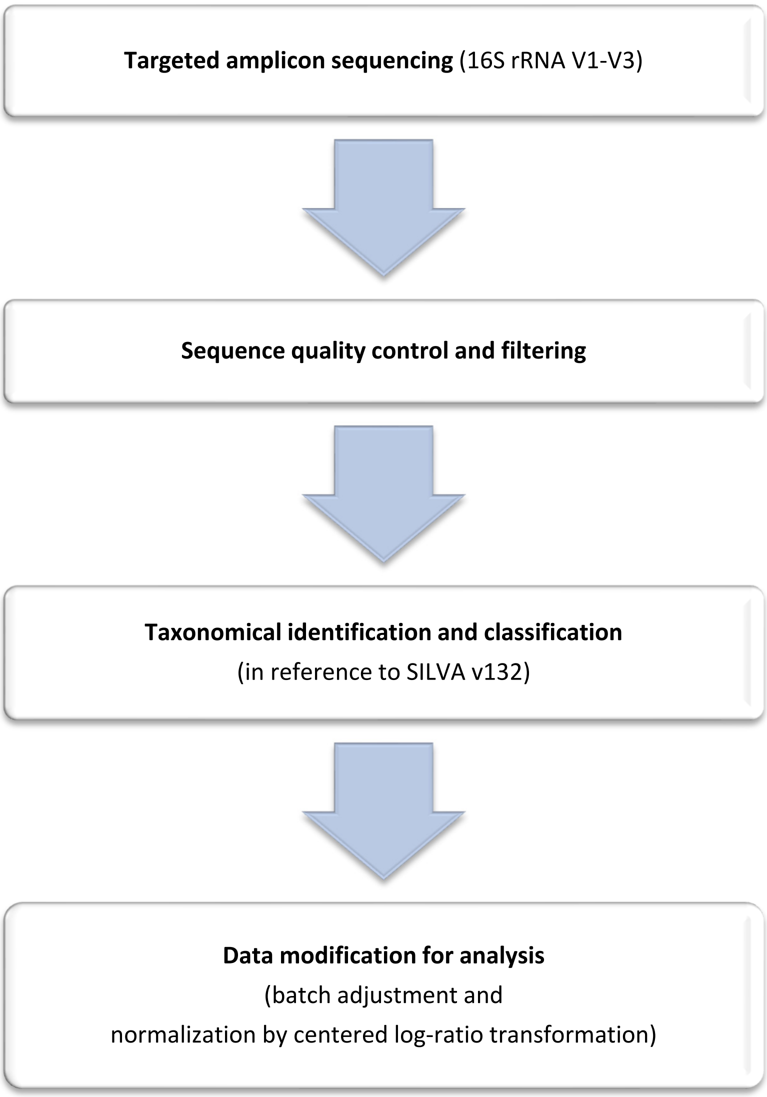

Figure S2. Dependency graph showing the links between processing steps, source files, and output files for the Multiethnic Cohort Study 16S datafiles after OTU generation [16].

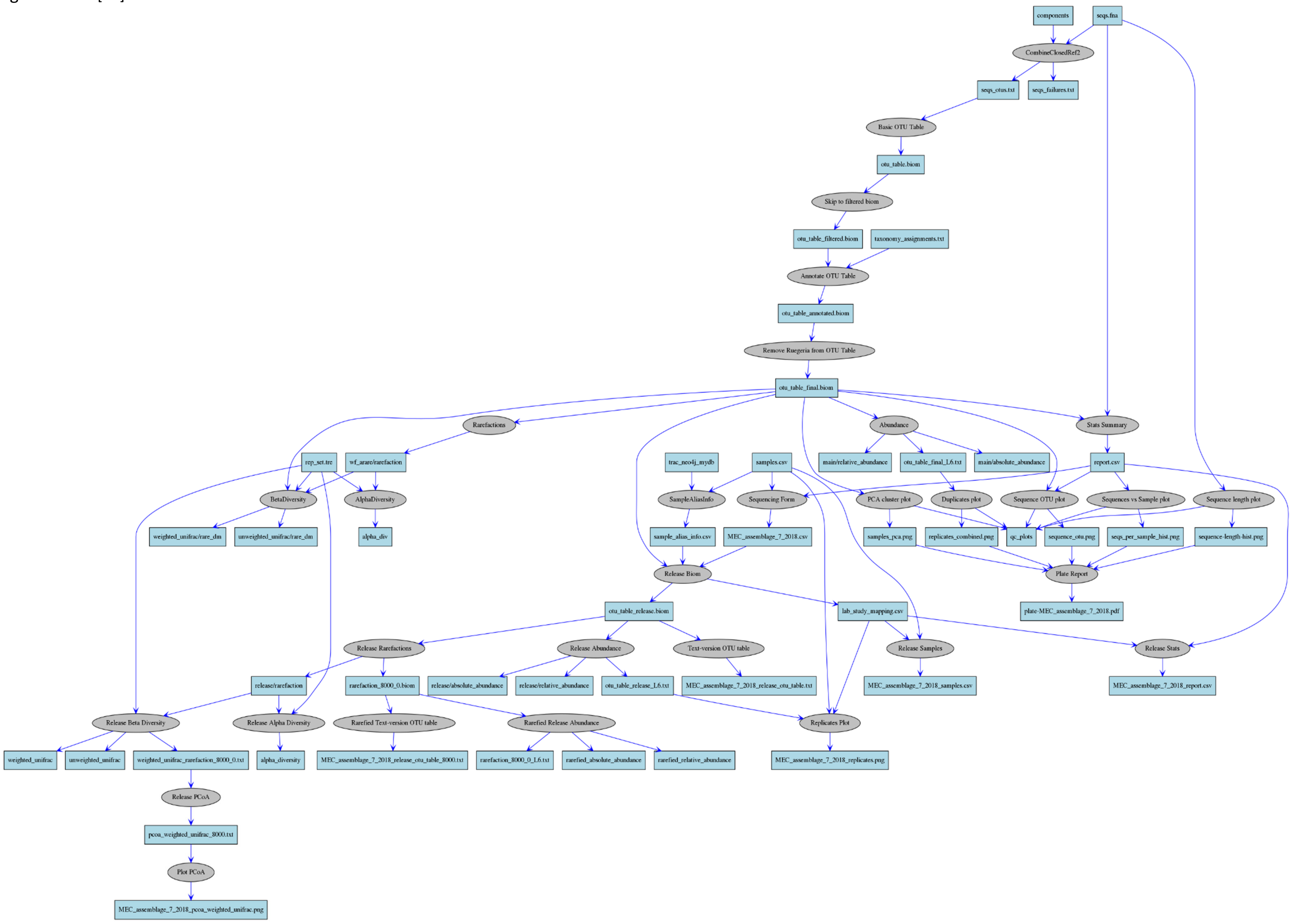

Figure S3. Participant Flow Chart.

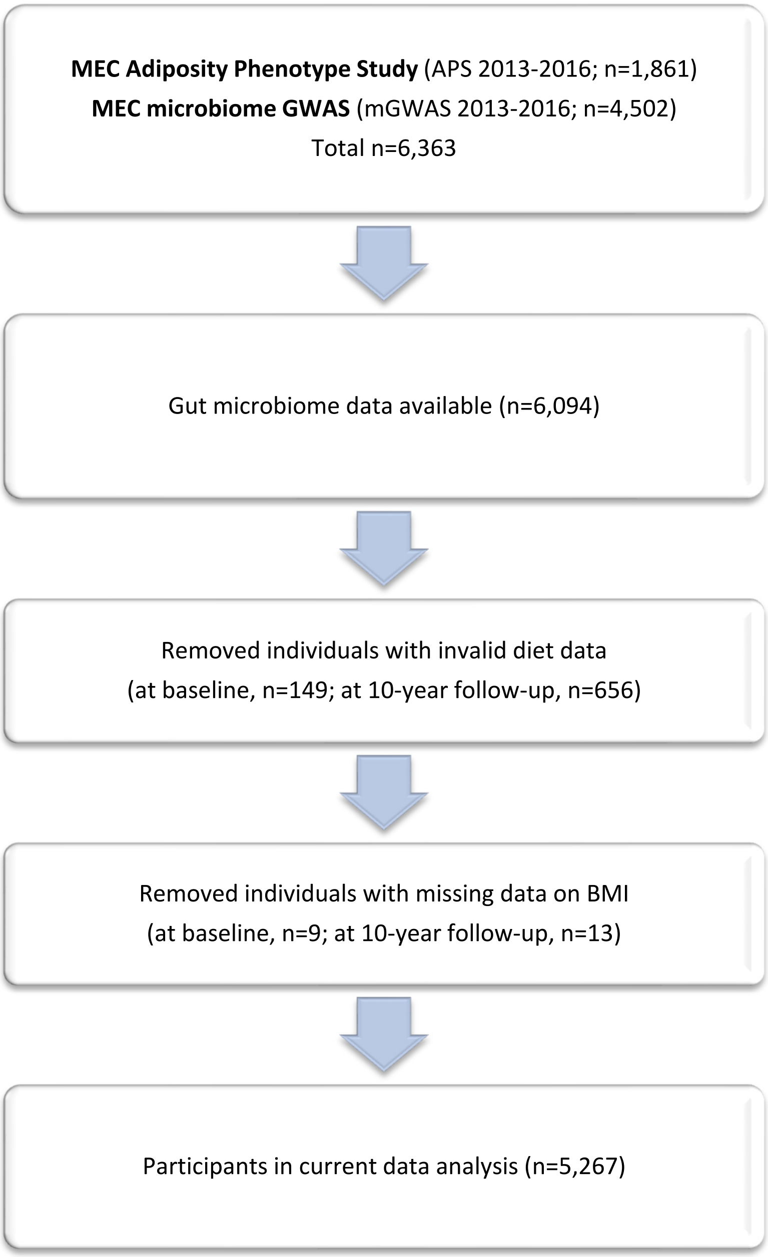

Figure S4. Conceptual framework for the current mediation analysis. The directed acyclic graph shows that the overall diet quality or its component foods/nutrients mediate (i.e., indirect effect) some of the ethnic differences in the gut microbial composition independently of confounders (age, sex, energy intake, body mass index (BMI) and antibiotics use).

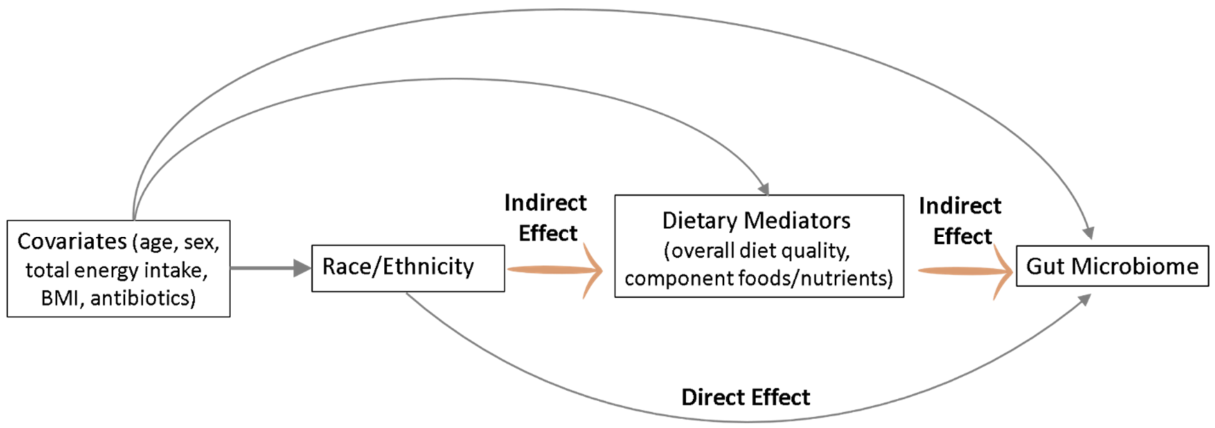

Figure S5. Spearman correlations between 15 dietary factors and 12 gut microbial genera that showed significant mediation. The heatmap indicates the correlation coefficients for positive (green) or negative (pink) correlations.

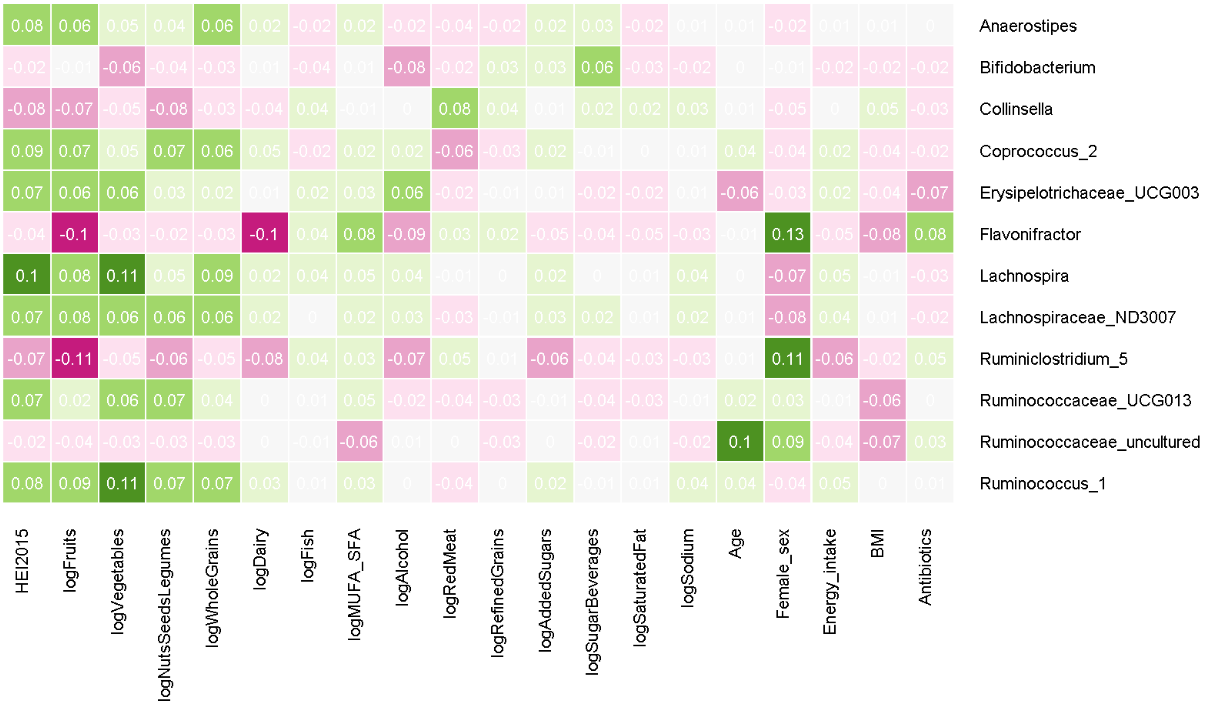

Table S1. Description (mean (standard deviation)) of gut microbial diversity and phyla abundance, overall and by race/ethnicity.

	**Overall**	**White**	**African American**	**Native Hawaiian**	**Japanese American**	**Latino**
N	5,267	918	750	684	1969	946
Alpha diversity (batch-adjusted)						
Chao1 index	1055 (138)	1057 (137)	1063 (131)	1078 146)	1042 (143)	1057 (122)
Phylogenetic diversity	24.3 (3.6)	24.5 (3.7)	24.9 (3.3)	25.0 (4.1)	23.5 (3.7)	24.6 (3.0)
Shannon index	6.52 (0.72)	6.68 (0.68)	6.63 (0.72)	6.63 (0.70)	6.29 (0.72)	6.68 (0.63)
Phyla (batch-adjusted, CLR-transformed)						
Actinobacteria	0.02 (3.63)	-0.06 (1.24)	0.06 (1.23)	0.23 (1.26)	-0.11 (1.39)	-0.03 (1.13)
Bacteroidetes	4.18 (0.90)	4.13 (0.86)	4.10 (0.96)	4.20 (0.93)	4.27 (0.89)	4.11 (0.89)
Cyanobacteria	-0.86 (1.75)	-0.99 (1.78)	-0.92 (1.77)	-0.93 (1.82)	-0.75 (1.71)	-0.88 (1.73)
Firmicutes	4.50 (0.90)	4.50 (0.85)	4.48 (0.91)	4.54 (0.93)	4.51 (0.92)	4.47 (0.86)
Fusobacteria	-2.19 (2.18)	-2.40 (2.12)	-1.82 (2.20)	-2.39 (2.13)	-2.32 (2.16)	-1.86 (2.23)
Lentisphaerae	-1.22 (1.82)	-1.45 (1.83)	-1.24 (1.81)	-1.41 (1.88)	-1.00 (1.79)	-1.33 (1.79)
Proteobacteria	0.84 (1.56)	0.64 (1.43)	0.49 (1.56)	1.10 (1.52)	1.04 (1.57)	0.70 (1.56)
Synergistetes	-1.37 (1.98)	-1.40 (1.92)	-1.61 (2.07)	-1.51 (2.03)	-1.22 (1.94)	-1.37 (1.96)
Tenericutes	-2.35 (2.19)	-1.94 (2.13)	-2.27 (2.10)	-2.37 (2.19)	-2.51 2.25)	-2.44 (2.14)
Verrucomicrobia	-1.51 (2.06)	-1.03 (2.01)	-1.27 (1.96)	-1.47 (2.06)	-1.91 (2.08)	-1.37 (1.99)

Table S2. Decomposition* of total ethnicity effect on 63 common genera abundance into direct effects and indirect effects mediated through dietary factors, adjusted for age, sex, energy intake, BMI and antibiotics use.

Genus	Foods	Reference	Comparison	Total Effect		Direct Effect		Indirect Effect (Mediation)			Percent Total Effect Mediated
				beta	SE	beta	SE	beta	SE	p-indirect	
Anaerostipes	HEI-2015	African Am.	Japanese Am.	-0.2339	0.0379	-0.2152	0.0381	-0.0187	0.0043	1.21E-5	8%
	Vegetables	Native Haw.	African Am.	-0.2453	0.0479	0.2742	0.0482	-0.0289	0.0062	3.44E-6	-12%
			Latino	0.1646	0.0452	0.1934	0.0444	-0.0288	0.0055	1.71E-7	-17%
Bifidobacterium	Dairy	White	Japanese Am.	0.4960	0.0611	0.5962	0.0657	-0.1002	0.0225	8.64E-6	-20%
	Alcohol	Japanese Am.	White	-0.4960	0.0611	-0.4160	0.0647	-0.0800	0.0176	5.75E-6	16%
	Added sugars	Japanese Am.	White	-0.4960	0.0611	-0.5419	0.0615	0.0459	0.0101	5.82E-6	-9%
Colinsella	Nuts/seeds/legumes	White	Native Haw.	0.4840	0.0717	0.4405	0.0715	0.0435	0.0096	5.29E-6	9%
Coprococcus-2	HEI-2015	African Am.	Japanese Am.	-0.2889	0.0605	-0.2599	0.0599	-0.0290	0.0067	1.33E-5	10%
	Nuts/seeds/legumes	White	Native Haw.	-0.2228	0.0660	-0.1868	0.0664	-0.0360	0.0081	9.80E-6	16%
	Red meat	African Am.	Native Haw.	-0.1718	0.0726	-0.1151	0.0731	-0.0567	0.0130	1.24E-5	33%
			Japanese Am.	-0.2889	0.0605	-0.2307	0.0620	-0.0582	0.0131	8.95E-6	20%
Erysipelotrichaceae UCG003	HEI-2015	African Am.	Latino	-0.2879	0.0886	-0.2334	0.0873	-0.0545	0.0125	1.32E-5	19%
Flavonifractor	HEI-2015	African Am.	Latino	-0.1896	0.0737	-0.2329	0.0751	0.0433	0.0090	1.64E-6	-23%
	Vegetables	Native Haw.	African Am.	-0.4547	0.0713	-0.4928	0.0705	0.0381	0.0080	2.17E-6	-8%
			Latino	-0.6443	0.0714	-0.6822	0.0700	0.0379	0.0078	1.17E-6	-6%
Lachnospira	Vegetables	Native Haw.	African Am.	-0.1964	0.0893	-0.1249	0.0912	-0.0715	0.0126	1.23E-8	36%
			Latino	-0.1826	0.0834	-0.1114	0.0835	-0.0712	0.0126	1.59E-8	39%
	Red meat	African Am.	Native Haw.	0.1964	0.0893	0.2773	0.0889	-0.0809	0.0170	1.91E-6	-41%
Lachnospiraceae ND3007	HEI-2015	African Am.	Japanese Am.	-0.4613	0.0618	-0.4317	0.0623	-0.0296	0.0068	1.24E-5	6%
	Vegetables	Native Haw.	Latino	0.2766	0.0704	0.3178	0.0704	-0.0412	0.0091	5.78E-6	-15%
	Saturated fat	Japanese Am.	White	0.2980	0.0594	0.3447	0.0602	-0.0467	0.0094	7.02E-7	-16%
Ruminiclostridium-5	HEI-2015	African Am.	Japanese Am.	0.4366	0.0364	0.4156	0.0374	0.0210	0.0046	4.71E-6	5%
			Latino	-0.1711	0.0371	-0.2046	0.0383	0.0335	0.0059	1.25E-8	-20%
	Vegetables	Native Haw.	African Am.	-0.2577	0.0387	-0.2843	0.0392	0.0266	0.0054	9.49E-7	-10%
			Latino	-0.4288	0.0396	-0.4553	0.0405	0.0265	0.0054	1.10E-6	-6%
	Red meat	African Am.	Native Haw.	0.2577	0.0387	0.2189	0.0401	0.0388	0.0084	3.61E-6	15%
			Japanese Am.	0.4366	0.0364	0.3968	0.0381	0.0398	0.0083	1.54E-6	9%
Ruminococcaceae UCG013	Nuts/seeds/legumes	White	Native Haw.	-0.1364	0.0612	-0.0933	0.0604	-0.0431	0.0089	1.14E-6	32%
			Latino	-0.2038	0.0561	-0.1694	0.0551	-0.0344	0.0071	1.45E-6	17%
Ruminococcaceae uncultured	HEI-2015	African Am.	Japanese Am.	-0.1989	0.0542	-0.2208	0.0540	0.0219	0.0050	1.04E-5	-11%

			Latino	-0.1783	0.0610	-0.2132	0.0614	0.0349	0.0069	3.93E-7	-20%
	Saturated fat	Japanese Am.	White	0.3241	0.0436	0.2885	0.0433	0.0356	0.0074	1.67E-6	11%
Ruminococcus-1	HEI-2015	African Am.	Japanese Am.	-0.2808	0.0404	-0.2576	0.0411	-0.0231	0.0050	3.31E-6	8%
	Vegetables	Native Haw.	African Am.	0.1299	0.0551	0.1748	0.0559	-0.0449	0.0082	5.21E-8	-35%
			Latino	0.1043	0.0478	0.1490	0.0482	-0.0447	0.0075	2.13E-9	-43%
	Red meat	African Am.	Native Haw.	-0.1299	0.0551	-0.0828	0.0569	-0.0471	0.0097	1.30E-6	36%
			Japanese Am.	-0.2807	0.0404	-0.2324	0.0424	-0.0483	0.0091	1.12E-7	17%

* The table shows the associations of race/ethnicity (total, direct, indirect) with only select gut microbial genera, where significant mediation through dietary quality-related factors was observed in the main analysis using past diet.

Table S3. Decomposition of total ethnicity effect on gut microbial genera into direct effects and indirect effects mediated through dietary factors, adjusted for overall diet quality (HEI-2015) as well as age, sex, energy intake, BMI and antibiotics use.

				Total Effect		Direct Effect		Indirect Effect (Mediation)			Percent Total Effect Mediated (without HEI adjustment)
Genus	Foods	Reference	Comparison	beta	SE	beta	SE	beta	SE	p-indirect	
Anaerostipes	Vegetables	Native Haw.	African Am.	-0.2245	0.0477	0.2513	0.0480	-0.0269	0.0067	6.12E-5	12% (12%)
			Latino	0.1736	0.0449	0.1915	0.0444	-0.0179	0.0044	4.89E-5	-10% (-17%)
Bifidobacterium	Dairy	White	Japanese Am.	0.4895	0.0612	0.5982	0.0659	-0.1087	0.0216	5.03E-7	-22% (-20%)
	Alcohol	Japanese Am.	White	-0.4895	0.0612	-0.4116	0.0647	-0.0779	0.0172	6.04E-6	16% (16%)
	Added sugars	Japanese Am.	White	-0.4895	0.0612	-0.5379	0.0618	0.0484	0.0111	1.36E-5	-10% (-9%)
Colinsella	Nuts/seeds/legumes	White	Native Haw.	0.4623	0.0712	0.4321	0.0713	0.0301	0.0083	0.00028	7% (9%)
Coprococcus-2	Nuts/seeds/legumes	White	Native Haw.	-0.1950	0.0667	-0.1742	0.0666	-0.0208	0.0068	0.0021	11% (16%)
	Red meat	African Am.	Native Haw.	-0.1395	0.0710	-0.1134	0.0718	-0.0261	0.0111	0.018	19% (33%)
			Japanese Am.	-0.2599	0.0599	-0.2325	0.0614	-0.0274	0.0115	0.017	11% (20%)
Flavonifractor	Vegetables	Native Haw.	African Am.	-0.4245	0.0713	-0.4574	0.0719	0.0329	0.0102	0.0013	-8% (-8%)
			Latino	-0.6573	0.0705	-0.6793	0.0694	0.0219	0.0068	0.0014	-3% (-6%)
Lachnospira	Vegetables	Native Haw.	African Am.	-0.2481	0.0884	-0.1817	0.0913	-0.0664	0.0132	4.72E-7	27% (36%)
			Latino	-0.1603	0.0829	-0.1160	0.0830	-0.0443	0.0102	1.43E-5	28% (39%)
	Red meat	African Am.	Native Haw.	0.2481	0.0884	0.2801	0.0888	-0.020	0.0142	0.025	-13% (-41%)
Lachnospiraceae ND3007	Vegetables	Native Haw.	Latino	0.2908	0.0708	0.3147	0.0705	-0.0239	0.0076	0.0017	-8% (-15%)
	Saturated fat	Japanese Am.	White	0.2731	0.0600	0.3056	0.0612	-0.0326	0.0115	0.0045	-12% (-16%)
Ruminiclostridium-5	Vegetables	Native Haw.	African Am.	-0.2343	0.0394	-0.2553	0.0413	0.0211	0.0064	0.0010	-9% (-10%)
			Latino	-0.4389	0.0400	-0.4529	0.0408	0.0140	0.0047	0.0027	-3% (-6%)
	Red meat	African Am.	Native Haw.	0.2343	0.0394	0.2176	0.0402	0.0166	0.0072	0.021	7% (15%)
			Japanese Am.	0.4156	0.0374	0.3982	0.0383	0.0175	0.0073	0.016	4% (9%)
Ruminococcaceae UCG013	Nuts/seeds/legumes	White	Native Haw.	-0.1169	0.0615	-0.0862	0.0606	-0.0307	0.0075	4.61E-5	26% (32%)
			Latino	-0.1744	0.0553	-0.1543	0.0546	-0.0201	0.0054	0.00021	12% (17%)
Ruminococcaceae uncultured	Saturated fat	Japanese Am.	White	0.3425	0.0436	0.3167	0.0433	0.0258	0.0090	0.0043	8% (11%)
Ruminococcus-1	Vegetables	Native Haw.	African Am.	0.1042	0.0554	0.1518	0.0573	-0.0476	0.0092	2.36E-7	-46% (-35%)
			Latino	0.1154	0.0477	0.1471	0.0482	-0.0317	0.0067	2.05E-6	-27% (-43%)
	Red meat	African Am.	Native Haw.	-0.1042	0.0554	-0.0815	0.0568	-0.027	0.0084	0.0069	22% (36%)
			Japanese Am.	-0.2576	0.0411	-0.2338	0.0425	-0.0238	0.0083	0.0040	9% (17%)

Table S4. Mean relative abundance of 12 genera most mediated by dietary factors, stratified by race/ethnicity.

Genus	White (n=918)	African American (n=750)	Native Hawaiian (n=684)	Japanese American (n=1969)	Latino (n=946)	p ethnicity
Anaerostipes	1.88 (1.74, 2.03)	2.12 (1.98, 2.27)	1.89 (1.74, 2.03)	1.89 (1.76, 2.03)	2.05 (1.91, 2.19)	1.3E-06
Bifidobacterium	-0.93 (-1.18, -0.68)	-0.45 (-0.70, -0.19)	-0.66 (-0.92, -0.40)	-0.43 (-0.67, -0.19)	-0.49 (-0.74, -0.24)	3.2E-08
Collinsella	0.80 (0.58, 1.01)	0.88 (0.66, 1.10)	1.28 (1.06, 1.50)	0.97 (0.77, 1.18)	0.81 (0.59, 1.02)	1.3E-12
Coprococcus-2	0.80 (0.60, 1.00)	0.74 (0.54, 0.95)	0.58 (0.37, 0.79)	0.46 (0.26, 0.65)	0.78 (0.58, 0.98)	2.2E-08
Erysipelotrichaceae UCG003	0.29 (-0.01, 0.59)	0.45 (0.16, 0.75)	0.15 (-0.15, 0.46)	0.23 (-0.05, 0.51)	0.17 (-0.12, 0.46)	0.013
Flavonifractor	-0.53 (-0.74, -0.31)	-0.67 (-0.89, -0.46)	-0.23 (-0.45, -0.02)	0.39 (0.19, 0.60)	-0.88 (-1.09, -0.67)	1.6E-81
Lachnospira	0.11 (-0.14, 0.36)	-0.05 (-0.30, 0.20)	0.17 (-0.09, 0.42)	0.08 (-0.16, 0.31)	-0.02 (-0.27, 0.22)	0.035
Lachnospiraceae ND3007	0.33 (0.10, 0.55)	0.48 (0.25, 0.70)	0.18 (-0.05, 0.41)	0.02 (-0.19, 0.23)	0.45 (0.23, 0.67)	7.6E-12
Ruminiclostridium-5	1.62 (1.49, 1.75)	1.53 (1.40, 1.66)	1.78 (1.64, 1.91)	1.96 (1.84, 2.09)	1.35 (1.22, 1.48)	5.5E-56
Ruminococcaceae UCG013	0.57 (0.36, 0.78)	0.39 (0.18, 0.60)	0.43 (0.21, 0.64)	0.53 (0.33, 0.73)	0.36 (0.15, 0.56)	0.0039
Ruminococcaceae uncultured	0.34 (0.17, 0.52)	0.22 (0.05, 0.40)	0.23 (0.05, 0.40)	0.02 (-0.14, 0.19)	0.04 (-0.13, 0.21)	5.7E-08
Ruminococcus-1	2.50 (2.35, 2.66)	2.69 (2.53, 2.84)	2.57 (2.42, 2.73)	2.41 (2.27, 2.55)	2.68 (2.53, 2.82)	0.0084

The mean genus proportion (95% confidence limit) for each of the 12 food-mediated genera was obtained from a general linear model of the genus in CLR values on ethnicity, adjusted for age, sex, BMI and antibiotics use. The p-value for ethnic differences was obtained from median regression.

Table S5. Decomposition* of total ethnicity effect on the 12 genera abundance from the main analysis (Table S2) into direct effects and indirect effects mediated through dietary factors using the concurrent Food Frequency Questionnaire data available in the Adiposity Phenotype Study subset (n=1,484), adjusted for age, sex, energy intake, BMI and antibiotics use.

Genus	Foods	Reference	Comparison	Total Effect		Direct Effect		Indirect Effect (Mediation)			Percent Total Effect Mediated
				beta	SE	beta	SE	beta	SE	p-indirect	
Anaerostipes	HEI-2015	African Am.	Japanese Am.	-0.2247	0.0827	-0.2037	0.0831	-0.0211	0.0093	0.024	9%
	Vegetables	Native Haw.	African Am.	0.2682	0.0839	0.2863	0.0837	-0.0182	0.0107	0.090	-7%
			Latino	0.2289	0.0851	0.2511	0.0843	-0.0221	0.0097	0.022	-10%
Bifidobacterium	Dairy	White	Japanese Am.	0.3220	0.1010	0.4086	0.1112	-0.0865	0.0388	0.026	-27%
	Alcohol	Japanese Am.	White	-0.3220	0.1010	-0.2667	0.1023	-0.0553	0.0337	0.100	17%
	Added sugars	Japanese Am.	White	-0.3220	0.1010	-0.3303	0.1048	0.0083	0.0139	0.550	-3%
Colinsella	Nuts/seeds/legumes	White	Native Haw.	0.3649	0.1174	0.3380	0.1177	0.0269	0.0123	0.029	7%
Coprococcus-2	HEI-2015	African Am.	Japanese Am.	-0.4310	0.1287	-0.3867	0.1286	-0.0442	0.0189	0.019	10%
	Nuts/seeds/legumes	White	Native Haw.	-0.4343	0.1040	-0.4169	0.1029	-0.0173	0.0129	0.180	4%
	Red meat	African Am.	Native Haw.	-0.2634	0.1239	-0.1828	0.1274	-0.0807	0.0252	0.001	31%
			Japanese Am.	-0.4341	0.1287	-0.3483	0.1330	-0.0858	0.0275	0.002	20%
Erysipelotrichaceae UCG003	HEI-2015	African Am.	Latino	-0.1575	0.1789	-0.1175	0.1793	-0.0400	0.0190	0.035	25%
Flavonifractor	HEI-2015	African Am.	Latino	-0.3519	0.1289	-0.3836	0.1297	0.0317	0.0137	0.020	-9%
	Vegetables	Native Haw.	African Am.	-0.1760	0.1407	-0.1990	0.1415	0.0230	0.0151	0.128	-13%
			Latino	-0.5265	0.1124	-0.5545	0.1129	0.0280	0.0138	0.042	-5%
Lachnospira	Vegetables	Native Haw.	African Am.	-0.2788	0.1714	-0.2433	0.1711	-0.0356	0.0203	0.079	13%
			Latino	-0.2301	0.1413	-0.1867	0.1393	-0.0433	0.0177	0.014	19%
	Red meat	African Am.	Native Haw.	0.2788	0.1714	0.2987	0.1758	-0.0199	0.0269	0.460	-7%
Lachnospiraceae ND3007	HEI-2015	African Am.	Japanese Am.	-0.5314	0.1253	-0.4972	0.1258	-0.0342	0.0154	0.026	6%
	Vegetables	Native Haw.	Latino	0.4590	0.1258	0.4915	0.1241	-0.0326	0.0161	0.044	-7%
	Saturated fat	Japanese Am.	White	0.3834	0.1032	0.3897	0.1036	-0.063	0.0099	0.529	-2%
Ruminiclostridium-5	HEI-2015	African Am.	Japanese Am.	0.4503	0.0859	0.4207	0.0837	0.0296	0.0105	0.005	7%
			Latino	-0.1522	0.0854	-0.1834	0.0837	0.0312	0.0124	0.012	-21%
	Vegetables	Native Haw.	African Am.	-0.2231	0.0867	-0.2328	0.0858	0.0096	0.0069	0.163	-4%
			Latino	-0.3726	0.0660	-0.3843	0.0667	0.0117	0.0064	0.067	-3%
	Red meat	African Am.	Native Haw.	0.2231	0.0867	0.1772	0.0869	0.0460	0.0157	0.003	21%
			Japanese Am.	0.4588	0.0822	0.4099	0.0856	0.0489	0.0167	0.003	11%
Ruminococcaceae UCG013	Nuts/seeds/legumes	White	Native Haw.	-0.1327	0.0984	-0.0904	0.0945	-0.0423	0.067	0.011	32%
			Latino	-0.1659	0.0856	-0.1267	0.0843	-0.0393	0.0141	0.006	24%

Ruminococcaceae uncultured	HEI-2015	African Am.	Japanese Am.	-0.3080	0.1105	-0.3284	0.1119	0.0205	0.0097	0.035	-7%
			Latino	-0.1004	0.1175	-0.1219	0.1182	0.0216	0.0117	0.067	-21%
	Saturated fat	Japanese Am.	White	0.5490	0.0844	0.5433	0.0837	0.0058	0.0090	0.519	1%
Ruminococcus-1	HEI-2015	African Am.	Japanese Am.	-0.3601	0.0809	-0.3284	0.0818	-0.0318	0.0124	0.010	9%
	Vegetables	Native Haw.	African Am.	0.1280	0.0986	0.1514	0.1000	-0.0234	0.0140	0.093	-18%
			Latino	0.1620	0.0855	0.1905	0.0868	-0.0285	0.0121	0.019	-18%
	Red meat	African Am.	Native Haw.	-0.1280	0.0986	-0.0902	0.1006	-0.0377	0.0189	0.046	29%
			Japanese Am.	-0.3521	0.0825	-0.3120	0.0867	-0.0401	0.0192	0.036	11%

* The table shows the associations of race/ethnicity (total, direct, indirect) with only select gut microbial genera, where significant mediation through dietary quality-related factors was observed in the main analysis using past diet. * The table shows the associations of race/ethnicity (total, direct, indirect) with only select gut microbial genera, where significant mediation through dietary quality-related factors was observed (Supplementary Table S2). Comparing the dietary mediation using the concurrent diet (Supplementary Table S5; n=1,484) vs. the past diet (Supplementary Table S2; n=5,267), we note that:

- for the 37 associations that showed significant past dietary mediation after adjusting for multiple tests (Supplementary Table S2), the direction of the dietary mediation (indirect effect), as well as the total and direct effects, was the same for all 37 associations in both data;
- of the 37 associations, 24 showed marginal significance (p-indirect effect <0.05) in the smaller concurrent diet data (Supplementary Table S5);
- the percent mediated (i.e., % of total race/ethnicity effect on each genus that is mediated by a given dietary factor) was about the same for 18 associations (absolute difference ≤5%; shaded in light orange) and further strengthened for an additional 4 associations (increased % positive/negative mediation by >5%; shaded in darker orange) using concurrent diet vs. the past diet data;
- and, of the other 15 associations that showed weakened mediation using concurrent diet (decreased % positive/negative mediation by >5%; shaded in blue), 7 associations still showed marginally significant mediation by diet (p-indirect effect <0.05).

Therefore, the past and concurrent diet data show some consistencies in supporting dietary mediation of the racial/ethnic differences in these gut microbial genera.

Table S6. Comparison* of 15 dietary factors and 12 genera by study area within the same race/ethnicity of Whites or Japanese Americans.

	Whites			Japanese Americans		
	in Hawaii (n=840)	in California (n=78)	p	in Hawaii (n=1800)	in California (n=169)	p
**Dietary factors**						
HEI-2015	72.8 (72.2, 73.5)	73.1 (70.9, 75.3)	0.79	71.5 (71.0, 72.0)	70.0 (68.4, 71.6)	0.075
Fruits (cups/day)	1.99 (1.89, 2.08)	2.05 (1.74, 2.37)	0.71	1.83 (1.77, 1.90)	1.68 (1.48, 1.88)	0.15
Vegetables (cups/day)	2.25 (2.16, 2.34)	2.23 (1.92, 2.54)	0.89	2.13 (2.07, 2.19)	2.13 (1.93, 2.32)	0.98
Nuts, seeds, legumes (oz/day)	1.30 (1.21, 1.39)	1.36 (1.07, 1.66)	0.69	1.10 (1.05, 1.16)	1.06 (0.89, 1.23)	0.60
Whole grains (oz/day)	1.87 (1.77, 1.96)	1.64 (1.33, 1.95)	0.17	**1.75 (1.69, 1.82)**	**1.43 (1.22, 1.63)**	**0.0030**
Dairy (cups/day)	1.48 (1.42, 1.55)	1.59 (1.37, 1.81)	0.34	0.83 (0.80, 0.86)	0.90 (0.80, 1.00)	0.19
Fish (oz/day)	0.88 (0.83, 0.94)	0.89 (0.71, 1.07)	0.96	**1.06 (1.02, 1.10)**	**0.83 (0.70, 0.95)**	**0.00059**
MUFA/SFA ratio	1.21 (1.20, 1.23)	1.22 (1.17, 1.27)	0.75	1.38 (1.37, 1.39)	1.37 (1.34, 1.41)	0.79
Alcohol (g/day)	13.3 (11.9, 14.7)	12.9 (8.2, 17.6)	0.87	4.67 (4.15, 5.19)	4.91 (3.22, 6.61)	0.79
Red meat (oz/day)	1.52 (1.44, 1.59)	1.39 (1.15, 1.64)	0.34	1.63 (1.58, 1.68)	1.50 (1.33, 1.66)	0.13
Refined grains (oz/day)	3.72 (3.58, 3.85)	3.64 (3.19, 4.09)	0.75	4.88 (4.76, 5.00)	4.82 (4.42, 5.21)	0.77
Added sugars (tsp/day)	9.53 (9.11, 9.95)	9.08 (7.69, 10.46)	0.54	7.27 (7.05, 7.49)	7.33 (6.62, 8.04)	0.87
Sugar-sweetened beverages (g/day)	87.5 (74.8, 100.1)	80.1 (38.2, 121.9)	0.74	73.4 (66.4, 80.5)	81.8 (58.6, 104.9)	0.50
Saturated fat (g/day)	22.3 (21.6, 23.0)	21.6 (19.2, 23.9)	0.57	17.8 (17.4, 18.2)	18.1 (16.9, 19.3)	0.66
Sodium (g/day)	3.04 (2.96, 3.12)	3.05 (2.78, 3.32)	0.95	2.97 (2.91, 3.03)	3.03 (2.83, 3.22)	0.57
**12 Genera with significant food mediation**						
Anaerostipes	1.91 (1.85, 1.97)	1.89 (1.69, 2.10)	0.86	1.91 (1.86, 1.95)	2.00 (1.85, 2.15)	0.24
Bifidobacterium	-0.75 (-0.85, -0.65)	-0.61 (-0.95, -0.27)	0.43	-0.26 (-0.34, -0.17)	0.02 (-0.24, 0.29)	0.052
Collinsella	1.07 (0.97, 1.16)	1.23 (0.92, 1.55)	0.32	1.25 (1.01, 1.47)	1.24 (1.01, 1.47)	0.97
Coprococcus-2	0.90 (0.81, 0.99)	0.74 (0.43, 1.05)	0.32	0.56 (0.50, 0.63)	0.65 (0.43, 0.86)	0.47
Erysipelotrichaceae UCG003	0.55 (0.41, 0.68)	0.84 (0.41, 1.27)	0.20	0.49 (0.40, 0.58)	0.79 0.49, 1.09)	0.056
Flavonifractor	-0.66 (-0.75, -0.56)	-0.52 (-0.83, -0.22)	0.41	**0.28 (0.22, 0.35)**	**0.03 (-0.18, 0.25)**	**0.030**
Lachnospira	**0.36 (0.26, .47)**	**-0.21 (-0.56, 0.13)**	**0.0017**	**0.26 (0.18, 0.34)**	**0.62 (0.36, 0.89)**	**0.0095**
Lachnospiraceae ND3007	0.50 (0.39, 0.60)	0.65 (0.32, 0.99)	0.37	**0.17 (0.10, 0.24)**	**0.58 (0.34, 0.82)**	**0.0012**
Ruminiclostridium-5	1.58 (1.53, 1.64)	1.70 (1.51, 1.89)	0.25	1.95 (1.90, 1.99)	1.81 (1.67, 1.95)	0.064
Ruminococcaceae UCG013	0.57 (0.48, 0.65)	0.63 (0.35, 0.91)	0.66	0.54 (0.47, 0.61)	0.62 (0.39, 0.85)	0.51
Ruminococcaceae uncultured	0.36 (0.29, 0.43)	0.48 (0.25, 0.72)	0.32	0.08 (0.03, 0.14)	0.15 (-0.03, 0.33)	0.49
Ruminococcus-1	2.61 (2.55, 2.68)	2.55 (2.34, 2.76)	0.58	**2.49 (2.44, 2.54)**	**2.85 (2.68, 3.03)**	**0.00012**

* The mean dietary factor (score or amounts) or genus abundance (CLR values) was obtained in a general linear model of each dietary factor or genus abundance on study site (Hawaii or Los Angeles, California) adjusted for age, sex, BMI and calories (for food intake trait) or antibiotics use (for genera).
